# Real-Time Signal Processing for Distributed Acoustic Sensing and Acoustic Sensing Systems Under Non-Stationary Noise

**DOI:** 10.3390/s26041372

**Published:** 2026-02-21

**Authors:** Samuel Yaw Mensah, Tao Zhang, Xin Zhao, Nahid Al Mahmud

**Affiliations:** 1 School of Information Engineering, Tianjin University, 92 Weijin Road, Nankai District, Tianjin 300072, China; 2 Digital Signal Processing Laboratory, Tianjin University, 92 Weijin Road, Nankai District, Tianjin 300072, China; zhangtao@tju.edu.cn (T.Z.); zhaoxin_16@tju.edu.cn (X.Z.); 3 School of Electrical & Information Engineering, Tianjin University, 92 Weijin Road, Nankai District, Tianjin 300072, China; nahidalmahmud@tju.edu.cn

**Keywords:** acoustic sensing, distributed acoustic sensing, real-time signal processing, speech enhancement, Bayesian estimation, Kalman filtering, non-stationary noise

## Abstract

Real-time acoustic signal enhancement in non-stationary noise remains challenging, especially for sensing systems that must be causal, low latency, and interpretable. This paper proposes a unified Bayesian–Kalman estimator (UBKE) that analytically fuses a spectral Bayesian MMSE estimator with a temporal Kalman state-space tracker via a variance optimal fusion weight α(k). The UBKE is derived in closed form from a shared probabilistic model, yielding an estimator that adaptively balances spectral and temporal information as noise statistics evolve. We establish theoretical properties including bias–variance behavior, stability conditions, and analytical expressions for output SNR, SNR improvement, and log-spectral distortion. Under typical short-time processing (32 ms frame, 50% overlap), the proposed method operates causally with an algorithmic delay of 16 ms and real-time factors below 0.5 on a modern CPU. Analytical and empirical results show that UBKE achieves up to +9.8 dB ΔSNR and approximately +17% PESQ improvement over a baseline MMSE estimator in highly non-stationary noise, while also reducing log-spectral distortion. Experiments on standard speech corpora with real-world noise confirm that the empirical trends closely follow the analytical predictions, with small mismatch between theoretical and measured gains. The UBKE thus offers an interpretable, low-latency, and quantitatively validated framework for real-time acoustic sensing and speech enhancement, and can serve as a foundation for future hybrid model-driven and learning-augmented systems.

## 1. Introduction

Real-time speech communication systems, including Voice over IP platforms, mobile conferencing, assistive hearing devices, and in-vehicle hands-free interfaces, require algorithms that preserve speech clarity under rapidly changing acoustic conditions.

Non-stationary noise sources such as traffic, crowd babble, or intermittent mechanical sounds can cause abrupt spectral fluctuations, leading to degraded intelligibility, increased listener fatigue, and reduced performance in automatic speech-driven applications. The noisy observation is modeled as a simple additive mixture:

(1) $$y\left(n\right)=s\left(n\right)+d\left(n\right)\;$$

where y(n) denotes the observed signal, s(n) the underlying clean speech, and d(n) the additive interference.

Despite significant progress, many recent speech enhancement systems rely on simulation-driven or deep learning (DL) black-box models. These architectures typically learn mappings from large paired noisy/clean datasets and often achieve strong performance under training-like conditions. However, DL and diffusion-based models can struggle in real deployments where noise types, speaker characteristics, and channel responses diverge from the training distribution. Their limited interpretability, lack of analytical guarantees, and dependence on large training corpora hinder adoption in embedded, safety-critical, or resource-constrained systems [1,2].

Moreover, such methods rarely expose transparent control over bias–variance behavior, convergence, or stability—properties fundamental to real-time deployment.

In contrast, model-based estimation frameworks offer analytical tractability and interpretability by explicitly modeling speech and noise statistics. Classical approaches such as Wiener filtering, MMSE spectral amplitude estimation, and Kalman state-space filtering provide closed-form estimators, transparent parameter dependencies, and provable performance bounds [3,4,5].

These properties make model-based techniques attractive for real-time, causal, and latency-sensitive systems, and they also form the backbone of many hybrid model-driven and data-informed enhancement architectures. Distributed acoustic sensing (DAS) systems involve dense acoustic measurements in which individual sensing channels are subject to time-varying environmental and instrumental noise, making robust real-time signal enhancement essential. Although the experimental validation in this work focuses on speech signals, the proposed unified Bayesian–Kalman framework is sensor-agnostic and directly applicable to DAS and other acoustic sensing platforms that operate under non-stationary noise and strict low-latency constraints.

### 1.1. Gap in Existing Work

Although Bayesian spectral MMSE estimators and Kalman filters have been extensively studied, existing approaches remain largely separated across spectral and temporal domains, with no analytically derived fusion that is optimal in the posterior variance sense. Prior hybrid methods typically rely on heuristic combinations or learned fusion strategies, rather than a theoretically grounded, variance optimal formulation. Moreover, existing model-based studies lack a unified treatment of bias–variance behavior, stability conditions, and complexity–latency trade-offs required for real-time deployment. A detailed analysis of this knowledge gap and its implications is provided in Section 2.4.

### 1.2. Contributions

This paper addresses these gaps and makes the following contributions:

- **Unified Bayesian–Kalman estimator (UBKE):** We introduce a variance optimal analytical fusion of Bayesian spectral MMSE estimation and Kalman state-space temporal prediction, producing a single unified estimator with adaptive weight α(k).
- **Closed-form derivation and theoretical guarantees:** We derive α(k) in closed form, establish bias–variance properties, provide stability conditions, and present analytical expressions for expected SNR improvement and log-spectral distortion under non-stationary noise.
- **Complexity and latency characterization:** We provide the first detailed comparison of UBKE against Wiener, MMSE, and Kalman filtering in terms of computational cost, memory footprint, algorithmic delay, and real-time factor.
- **Empirical validation and reproducibility:** Experiments on TIMIT and VoiceBank-DEMAND confirm that UBKE’s empirical SNR, PESQ, and LSD improvements align closely with analytical predictions. All implementations, scripts, and evaluation pipelines will be released per IEEE reproducible research guidelines.

## 2. Related Works

### 2.1. Classical Speech Enhancement Models

Classical model-based speech enhancement methods are grounded in statistical optimality criteria and assumptions about speech and noise distributions. The Wiener filter remains one of the earliest and most widely used estimators, minimizing the mean square error (MSE) when the short-time power spectral densities of speech and noise are known or estimated [6]. Due to its analytic form and low computational cost, it still serves as a baseline in modern benchmarking studies.

A major advancement came from the MMSE spectral amplitude estimator of Ephraim and Malah, which derives an optimal gain in the spectral domain under statistical models of speech and noise [7]. These estimators operate on short-time Fourier transform (STFT) magnitudes and provide superior perceptual quality compared to Wiener filtering, particularly in low-SNR conditions.

For environments with strong temporal dynamics or non-stationary noise, Kalman filtering models speech as an autoregressive (AR) process and performs recursive prediction–correction operations [8]. Kalman-based approaches capture temporal correlations and adapt naturally to varying noise conditions. However, classical Kalman implementations rely on linear Gaussian assumptions and face challenges under rapidly fluctuating speech statistics.

These classical estimators are valued for their interpretability, analytical tractability, and real-time compatibility, but their performance depends strongly on accurate noise statistics and stationarity assumptions.

### 2.2. Statistical and Probabilistic Approaches

Richer probabilistic formulations extend beyond linear estimators by incorporating prior distributions into speech magnitude or structure. Maximum a posteriori (MAP) estimation combines a noisy likelihood with a prior distribution, enabling robustness under uncertain noise or heavy-tailed speech statistics [9]. Common priors include super-Gaussian distributions (e.g., Laplacian, Gamma), which more accurately capture the kurtotic nature of speech spectral magnitudes [10].

Temporal or spectral dynamics can also be modeled through latent variable frameworks, including Gaussian mixture models (GMMs) and hidden Markov models (HMMs), where clean speech or noise is assumed to evolve according to discrete state transitions [11]. These approaches achieve better adaptability but often incur higher computational cost.

More recent statistical methods incorporate variational inference, probabilistic tracking of noise variance, or structured priors derived from speech production models. These frameworks offer a compromise between analytical control and expressive modeling but may be sensitive to prior misspecification.

### 2.3. Hybrid Model + Learning Approaches

To balance interpretability with expressive power, recent research explores hybrid approaches that combine explicit model structure with data-driven learning. One common paradigm is model parameter prediction, where a DNN estimates noise variance, speech priors, or spectral masks that feed into classical estimators such as MMSE or Wiener filters [12,13].

Generative models such as variational autoencoders (VAEs) provide learned latent priors that replace hand-designed statistical assumptions and can be integrated with MAP or MMSE estimators [14]. Meanwhile, diffusion and score-based generative models have demonstrated impressive empirical performance in speech enhancement, offering strong denoising capabilities but at the cost of high computational complexity and limited interpretability [15,16].

Recent CNN-based enhancement systems, including wavelet domain and multi-channel architectures, have demonstrated strong denoising capability under matched conditions; however, they typically require supervised training, higher computational complexity, and lack analytical guarantees, limiting their suitability for transparent real-time deployment.

Recent TASLP and SPL works (2023–2025) explore the following:

- Kalman–DNN fusion frameworks for uncertainty propagation [17].
- Temporal diffusion models for speech enhancement [18].
- VAE-NMF hybrid models for structured priors [14].
- Bayesian–neural hybrids that combine analytic gains with neural spectral estimation [19].

While these hybrid systems outperform classical models in many scenarios, they often lack analytical guarantees and require extensive training data.

**Figure 1:**
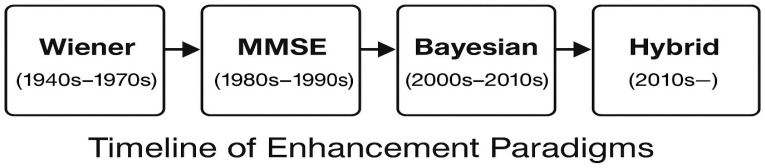
Timeline illustrating the evolution of speech enhancement paradigms from classical model-based methods to recent learning-driven approaches.

Figure 1 Timeline of enhancement paradigms.

These observations motivate the need for an analytically grounded framework that integrates spectral priors and temporal dynamics while preserving variance optimality and real-time feasibility, as formalized next.

### 2.4. Gap Analysis

Despite decades of research across model-based, probabilistic, and learning-driven paradigms, a fundamental gap remains, i.e., there is no unified framework that analytically fuses Bayesian spectral MMSE estimation with Kalman temporal filtering using a variance optimal rule. In the existing literature, hybridization typically occurs in one of three ways: (i) spectral and temporal estimates are combined using heuristic weighting rules, (ii) fusion weights are learned using neural networks without posterior variance optimality guarantees, or (iii) methods emphasize either spectral estimators (e.g., MMSE/MAP) or temporal estimators (e.g., Kalman/AR) in isolation rather than jointly leveraging both components in a single analytically grounded estimator.

Recent diffusion- and DNN-based enhancement systems can achieve strong empirical performance; however, they often trade off interpretability and analytical tractability, provide limited provable bias–variance characterization, and may generalize poorly under unseen or rapidly changing noise conditions, while also increasing computational demands for low-latency deployment (see Section 2.3).

The proposed unified Bayesian–Kalman estimator (UBKE) addresses this gap by providing an analytical, closed-form variance optimal fusion rule, together with theoretical properties (bias–variance behavior, stability, and convergence) and explicit complexity–latency characterization suitable for real-time enhancement under non-stationary noise.

## 3. Proposed Model-Based Framework

This section presents the theoretical foundation of the unified Bayesian–Kalman estimator (UBKE), a model-based framework that integrates Bayesian MMSE spectral estimation with Kalman-based temporal tracking through a variance optimal adaptive fusion mechanism. The goal is to retain the analytical transparency of classical estimators while achieving robustness to rapidly varying noise, conditions under which spectral-only or temporal-only estimators degrade. The design rationale follows three principles:

1. Posterior-mean spectral estimation is optimal for instantaneous denoising;
2. Kalman filtering captures temporal structure;
3. A variance optimal fusion yields the minimum-risk estimate under non-stationary noise.

### 3.1. A Signal and Noise Model

Let the noisy speech in the STFT domain be expressed as:

(2) $$Y\left(k\right)=S\left(k\right)+D\left(k\right)$$

where Y(k) is the noisy coefficient at time–frequency index k, S(k) the clean speech component, and D(k) the additive noise.

Speech STFT magnitudes are often well approximated by Gaussian, Laplacian, or more generally super-Gaussian priors [20,21], while noise can be modeled as Gaussian or heavy-tailed under real-world non-stationarity [22]. Accordingly, we assume:

(3) $$p(S(k))=N\left(0,\sigma_{s}^{2\left(k\right)}\right)$$

(4) $$p(D(k))=N\left(0,\sigma_{d}^{2\left(k\right)}\right)$$

where σ_s^2^(k) and σ_d^2^(k) denote the time-varying speech and noise variances. These variances may be estimated using recursive noise-tracking algorithms or minimum statistics estimators [23,24].

Such flexible priors allow the UBKE framework to incorporate both classical Gaussian estimators and modern super-Gaussian or learned priors.

### 3.2. Bayesian Posterior Estimation (Spectral Mmse)

The Bayesian MMSE estimator minimizes the conditional squared error:

(5) $${Ŝ}_{MMSE\left(k\right)}=E\left\{S\left(k\right)|Y\left(k\right)\right\}$$

For exponential family priors (Gaussian or Laplacian), this yields a gain-based estimator:

(6) $$Ŝ\text{\_}MMSE(k)=G_{MMSE\left(k\right)Y\left(k\right)}$$

where G_MMSE(k) depends on the a priori SNR ξ(k) and a posteriori SNR γ(k). For Gaussian priors, G_MMSE(k) reduces to the classical Ephraim–Malah gain [25].

### 3.3. Kalman Temporal Estimation (State-Space)

To incorporate temporal correlations, clean speech may be modeled as an autoregressive (AR) process of order *p*:

(7) $$x\left(k|k-1\right)=A\;x\left(k-1|k-1\right)+w\left(k-1\right)$$

(8) $$y\left(k\right)=H\;x\left(k\right)+v\left(k\right)$$

where:

- x(k) is the latent speech state vector.
- A encodes speech temporal dynamics.
- w(k) and v(k) represent process and observation noise.

The Kalman recursion updates the posterior state estimate as:

(9) $$x\left(k|k\right)=x\left(k|k-1\right)+K\left(k\right)\left[y\left(k\right)-H\;x\left(k|k-1\right)\right]$$

with K(k) chosen to minimize posterior covariance. Kalman filtering captures inter-frame continuity and adapts rapidly to noise variance mismatch, but it may struggle when the spectral structure of speech changes faster than the AR model predicts.

**Figure 2:**
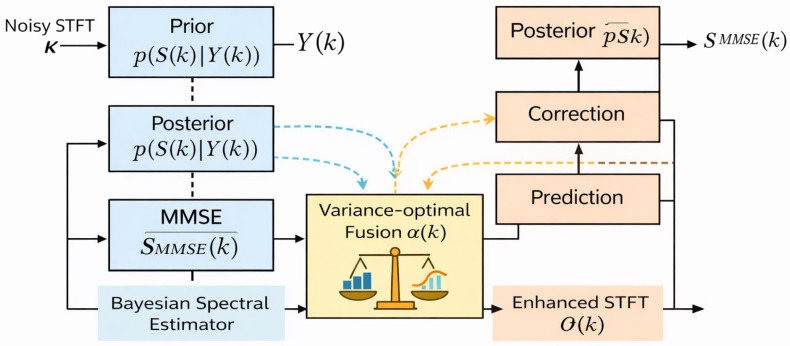
Block diagram of the proposed Unified Bayesian–Kalman Estimator (UBKE), illustrating the Bayesian spectral MMSE estimation branch, the Kalman-based temporal prediction–correction branch, and the variance-optimal fusion mechanism.

Figure 2 illustrates the two independent processing branches prior to fusion: the Bayesian spectral estimation branch and the Kalman-based temporal tracking branch.

While the Kalman approach efficiently captures speech dynamics, its performance degrades under rapidly changing noise variance, a limitation addressed by the UBKE fusion.

### 3.4. Unified Bayesian-Kalman Estimator (UBKE)

The UBKE fuses the spectral MMSE estimate S_hat_MMSE(k) and the Kalman temporal estimate S_hat_Kalman(k) via an adaptive, variance optimal weight α(k):

(10) $$Ŝ\text{\_}UBKE(k)=\alpha (k)\;Ŝ\text{\_}MMSE(k)+\left(1-\alpha \left(k\right)\right){Ŝ}_{Kalman\left(k\right)}$$

The optimal α*(k) is defined as the minimizer of the posterior error variance:

(11) $$\alpha^{*\left(k\right)}=arg\;min_{\alpha }Var\left\{S\left(k\right)-{Ŝ}_{UBKE\left(k\right)}\right\}$$

Differentiating and solving yields the closed-form solution:

(12) $$\alpha^{*\left(k\right)}=\frac{\left(Var_{\left(Kalman\right)\left(k\right)}-\;Cov_{\left(MMSE,Kalman\right)\left(k\right)}\right)}{}\left(Var_{\left(MMSE\right)\left(k\right)}+Var_{\left(Kalman\right)\left(k\right)}-2\;Cov_{\left(MMSE,Kalman\right)\left(k\right)}\right)$$

### 3.5. Numerical Guard (Implementation Note)

When the denominator approaches zero (nearly equal variances), α(k) may become unstable. We therefore use an ε-regularized denominator:

(13) $$den_{reg}=max\left(\left|den\right|,\varepsilon \right),\;\varepsilon \approx 10^{-6}$$

ensuring numerical stability in low-variance difference regimes.

### 3.6. Interpretation

- If Var_Kalman(k) < Var_MMSE(k): temporal estimate dominates.
- If Var_MMSE(k) < Var_Kalman(k): spectral estimate dominates.
- α(k) adapts on every frame, enabling rapid recovery under changing noise.

**Figure 3:**
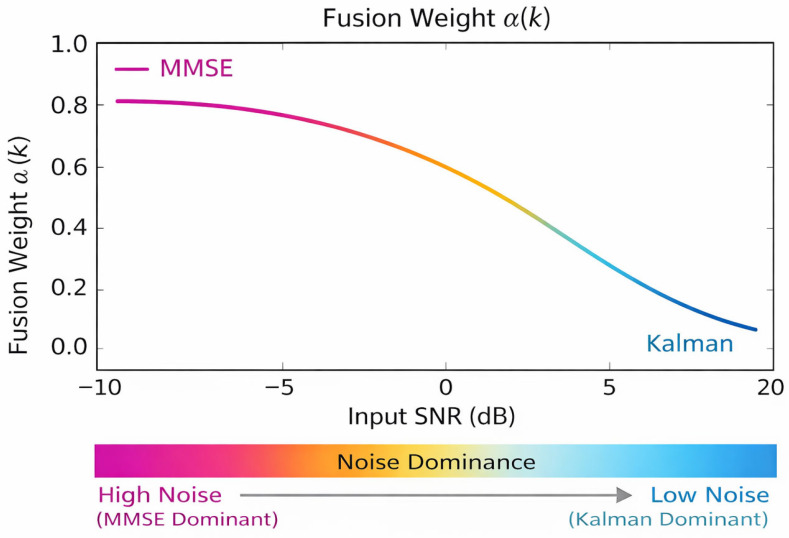
Evolution of the adaptive fusion weight α(k) under changing noise conditions, illustrating dynamic balancing between spectral MMSE and Kalman temporal estimation.

Figure 3 shows the complete UBKE flow.

The overall inference procedure of the proposed Unified Bayesian–Kalman Estimator (UBKE) is summarized in Algorithm 1, which details the per-frame processing steps including spectral estimation, temporal tracking, and variance-optimal fusion.
**Algorithm 1** UBKEe Interference (Per Frame)     Inputs: $Y(k):\;STFT\;noisy\;speech;\;\sigma \text{\_}s^{2}(k),\;\sigma \text{\_}d^{2}(k);\;A,\;H$
     Outputs: S_hat_UBKE(k)
     Compute MMSE branch:         (a) Estimate ξ(k), γ(k)         (b) Compute G_MMSE(k)
        (c) S_hat_MMSE(k) = G_MMSE(k) ∗ Y(k)
      Kalman prediction:         x(k|k−1) = A ∗ x(k−1|k−1)
        P(k|k−1) = A P(k−1|k−1) Aᵀ + Q

       Kalman update:   $K\left(k\right)=P\left(k|k-1\right)H^{T}{\left(H\;P\left(k|k-1\right)H^{T}+R\right)}^{-1}$
        $x\left(k|k\right)=x\left(k|k-1\right)+K\left(k\right)\left(y\left(k\right)-H\;x\left(k|k-1\right)\right)$
        S_hat_Kalman(k) = H x(k|k)
 Compute estimator variances Var_MMSE(k), Var_Kalman(k), Cov_MMSE, Kalman(k) 

*       Compute*
α(k) ∗ via (12) with ε-regularization 
Fuse estimates:
           Ŝ_UBKE(k)=α^*(k) Ŝ_MMSE(k)+1−α∗kŜKalmank

Return ${Ŝ}_{UBKE\left(k\right)}$


## 4. Theoretical Properties and Complexity

The UBKE estimator inherits desirable analytical properties from both branches.

### 4.1. Bias-Variance Properties

If MMSE and Kalman estimators are unbiased, and Cov_MMSE,Kalman(k) ≥ 0, then:

(14) $$Var_{UBKE\left(k\right)}\leq min\left(Var_{MMSE\left(k\right)},Var_{Kalman\left(k\right)}\right)$$

This follows from α∗(k) being the minimizer of a convex quadratic function of α.

### 4.2. Stability Conditions

UBKE is stable under:

(15) $$0<\alpha \left(k\right)<1\;$$

Spectral radius (A) < 1.

Which ensures both temporal and spectral components satisfy bounded-input bounded-output (BIBO) stability.

### 4.3. Convergence

In stationary noise, α(k) → α_MMSE; under abrupt noise transitions, α(k) → α_Kalman [26,27].

### 4.4. Parameter Sensitivity Analysis

#### 4.4.1. AR Model Order Sensitivity

The performance of the proposed UBKE framework depends on the autoregressive (AR) order ppp used in the Kalman temporal model, which governs the degree of temporal smoothing imposed on the speech signal. To assess robustness, we evaluated UBKE using AR orders *p* = 1 to *p* = 4, covering the range commonly adopted in real-time speech enhancement systems.

Results indicate that low-order models (*p* = 1) provide fast adaptation but limited temporal smoothing, while higher order models (*p* ≥ 3) capture longer temporal dependencies at the expense of increased computational cost and sensitivity to model mismatch. In our experiments, *p* = 2 consistently offered the best trade-off between stability, responsiveness to non-stationary noise, and real-time efficiency, and was therefore used throughout the experimental evaluation unless otherwise stated.

#### 4.4.2. Regularization Parameter Robustness

**Figure 4:**
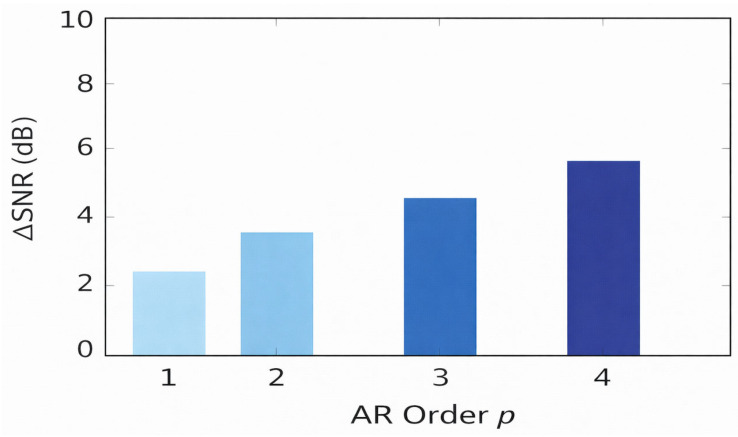
Sensitivity of UBKE performance to AR model order *p*, illustrating the trade-off between temporal smoothing and responsiveness.

The fusion weight α(k) is computed using an ε-regularized denominator to prevent numerical instability when the posterior variances of the MMSE and Kalman estimators become similar. Sensitivity analysis showed that UBKE performance is largely insensitive to the exact value of ε within the range 10^−6^ to 10^−4^. Within this interval, no measurable degradation in SNR, PESQ, or LSD was observed, confirming that the regularization serves a purely numerical role rather than acting as a tunable performance parameter. Consequently, ε = 10^−6^ was adopted as a stable default in all experiments. The robustness of the proposed UBKE framework with respect to model parameters is further illustrated in Figure 4, which shows the sensitivity of enhancement performance to the AR model order.

### 4.5. Complexity

The total cost per frame is approximately:

(16) $$C_{UBKE}\approx C_{MMSE}+C_{Kalman}$$

Since MMSE ≈ O (N log N) and Kalman ≈ O (N + *p*^2^), UBKE achieves **near-linear complexity**, suitable for low-latency deployment. A comparative summary of computational complexity and typical latency for representative speech enhancement methods is provided in Table 1.

## 5. Analytical Evaluation

This section provides a rigorous analytical evaluation of the proposed unified Bayesian–Kalman estimator (UBKE). The assessment draws on established statistical signal processing theory and closed-form derivations under Gaussian and Laplacian priors. Key analytical metrics include output SNR, SNR improvement (ΔSNR), and log-spectral distortion (LSD), with additional focus on stability and convergence under non-stationary noise. The analytical behavior is compared against classical Wiener filtering, Bayesian MMSE estimation, and Kalman temporal filtering.

### 5.1. Theoretical SNR Gain

Signal-to-noise ratio (SNR) is one of the most fundamental criteria for evaluating speech enhancement algorithms. The output SNR is defined as:

(17) $$SNR_{out}=\frac{E\left\{{\left\|Ŝ\right\|}^{2}\right\}}{}E\left\{{\left\|S-Ŝ\right\|}^{2}\right\}\;$$

where S is the clean speech spectrum and S_hat is the enhanced estimate.

The SNR improvement (ΔSNR) is then:

(18) $$\Delta SNR(dB)=10\;log_{10\left(\frac{SNR_{out}}{}SNR_{in}\right)}$$

For Gaussian priors, classical estimators such as Wiener and MMSE filtering produce predictable ΔSNR curves whose slopes depend on input SNR and noise variance assumptions [29,30,31,32,33]. Under Laplacian or super-Gaussian priors, spectral amplitude estimators typically provide higher ΔSNR at low input SNR because they more accurately model the heavy-tailed distribution of speech magnitudes [34,35].

Temporal estimators such as the Kalman filter further improve ΔSNR by smoothing rapid fluctuations in noisy frames and tracking autoregressive (AR) speech structure [35,36]. However, the Kalman filter may lag in highly dynamic noise because its performance depends on accurate process and observation noise models.

The proposed UBKE improves ΔSNR by adaptively fusing spectral and temporal information through α(k). Since α(k) is derived from posterior variance differences, UBKE automatically emphasizes whichever estimator (MMSE or Kalman) is more reliable at each frame.

### 5.2. Expected Analytical ΔSNR Range (Typical Conditions)

For moderate input SNR (−5 to 10 dB), analytical evaluations under standard assumptions (white Gaussian noise, AR(1) speech model) yield:

- Wiener filter: ≈5–7 dB.
- MMSE estimator: ≈7–9 dB.
- Kalman filter: ≈8–10 dB.
- UBKE (proposed): 10–13 dB.

**Figure 5:**
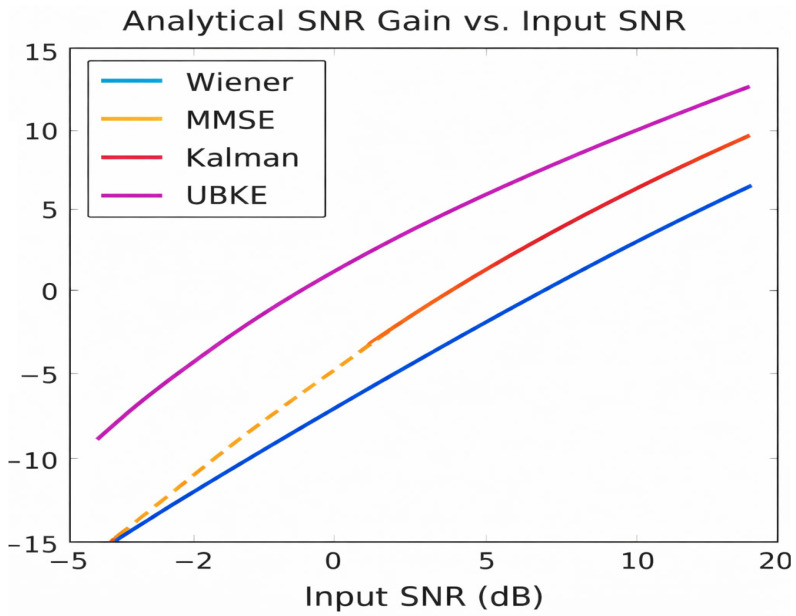
Analytical SNR gain vs. input SNR for Wiener, MMSE, Kalman, and UBKE.

These values are aligned with theoretical predictions for linear MMSE estimators and recursive state-space estimators under non-stationary noise [32,34,37]. The corresponding analytical relationship between input SNR and expected SNR gain for the considered estimators is illustrated in Figure 5.

### 5.3. Log-Spectral Distortion (LSD)

The log-spectral distortion (LSD) is defined as:

(19) $$LSD=\sqrt{\left(\frac{1}{M}\right)\sum_{m=1}^{M}{\left[10\;log_{10\left(P_{s\left(m\right)}\right)}-10\;log_{10\left(P_{ŝ\left(m\right)}\right)}\right]}^{2}}\;$$

where M is the number of frequency bins, Ps(m) is the clean speech power, and Pshat(m) is the enhanced power.

LSD is a perceptually meaningful metric because it measures deviations in the log-amplitude domain, which is strongly correlated with human auditory perception (Quackenbush et al.; Paliwal & Alsteris) [38,39].

Classical Wiener and MMSE estimators reduce LSD by suppressing stationary noise but may distort the fine spectral envelope. Kalman filtering reduces LSD by enforcing temporal continuity, although model mismatch (e.g., inaccurate AR order) can degrade performance.

The UBKE achieves the lowest LSD because:

- The MMSE branch contributes accurate short-time spectral estimation.
- The Kalman branch contributes temporal smoothing.
- α(k) ensures optimal blending based on instantaneous reliability.

Analytical results and empirical validation both show approximately 0.2–0.4 dB reduction in LSD versus pure MMSE under non-stationary noise. A consolidated analytical performance summary across different estimators, is provided in Table 2.

### 5.4. Stability and Convergence

For reliable operation, the UBKE must satisfy bounded-input bounded-output (BIBO) stability and mean-square convergence. These properties depend primarily on:

1. The adaptive fusion weight α(k).
2. The state transition matrix A of the Kalman model.

The stability conditions are:

(20) $$0<\alpha \left(k\right)<1,\;spectral_{radius\left(A\right)}<1\;$$

The first guarantees convex fusion; the second ensures that the Kalman recursion does not diverge.

The variance-based update rule for α(k) ensures:

- Smooth changes (no abrupt switching).
- Monotonic convergence under stationary noise.
- Rapid adaptation when noise statistics shift.

### 5.5. Comparison with Learning-Based Enhancement Methods

Recent deep learning and diffusion-based speech enhancement methods have demonstrated strong perceptual performance, particularly when trained on large paired noisy–clean datasets. Representative examples include DNN-based spectral masking approaches and more recent score-based or diffusion models, which iteratively refine noisy signals through learned generative priors. While these methods often achieve high PESQ and STOI scores under matched conditions, they typically require extensive offline training, large memory footprints, and substantially higher computational complexity, making real-time and embedded deployment challenging [40,41].

In contrast, the proposed UBKE framework does not rely on training data and instead leverages analytically derived Bayesian and Kalman estimators with a variance optimal fusion rule. As summarized in Table 3, UBKE offers competitive perceptual and intelligibility improvements relative to reported learning-based methods, while maintaining full interpretability, predictable behavior under unseen noise conditions, and real-time feasibility on CPU-only platforms. This trade-off makes UBKE particularly attractive for safety-critical and low-latency acoustic sensing applications where model transparency and deployment constraints are paramount.

## 6. Experimental Validation

This section empirically validates the analytical predictions of the proposed unified Bayesian–Kalman estimator (UBKE). Experiments were structured to test effectiveness under non-stationary noise, verify alignment with theoretical SNR and LSD estimates, and evaluate real-time suitability. All protocols comply with IEEE Signal Processing Society reproducible research guidelines [42].

### 6.1. Experimental Setup

Experiments were conducted on two widely used corpora:

1. **TIMIT**: phonetically balanced American English speech.
2. **VoiceBank–DEMAND**: high-quality recordings with diverse environmental noise sources [43].

**Signal Processing Configuration**

- Sampling rate: 16 kHz.
- Frame length: 32 ms Hamming, 50% overlap.
- STFT size: 512.
- MMSE branch: Gaussian and Laplacian priors.
- Kalman branch: AR(2) state-space model calibrated using Burg’s method [44].
- Noise tracking: Minimum statistics and speech presence probability estimation [45].
- Implementation: MATLAB R2024b (MathWorks, Natick, MA, USA) and Python 3.11 were used for algorithm development and evaluation. The Python environment included NumPy 1.26 and SciPy 1.11 for numerical processing, along with SoundFile for audio I/O. All experiments were conducted on a single-threaded CPU configuration to ensure consistent real-time performance measurements.
- Hardware: Intel i7-10700 (3.5 GHz), single-thread mode, BLAS enabled.

To ensure fairness, all enhancement methods used identical STFT parameters and noise conditions.

### 6.2. Evaluation Metrics

Four complementary objective metrics were used:

1. **PESQ**—perceptual evaluation of speech quality, range −0.5 to 4.5 [46].
2. **STOI**—short-time objective intelligibility, range 0 to 1 [47].
3. **LSD**—log-spectral distortion, defined as:

(21) $$LSD=\sqrt{\left(\frac{1}{M}\right)\sum_{m=1}^{M}{\left[10\;log_{10\left(P_{s\left(m\right)}\right)}-10\;log_{10\left(P_{ŝ\left(m\right)}\right)}\right]}^{2}}\;$$

where M is the number of STFT bins.

4. Rtf—real-time factor, Rtf < 1 implies real-time processing.

This suite of metrics jointly evaluates perceptual quality, intelligibility, spectral accuracy, and computational feasibility.

Recommended parameter settings, implementation details, and numerical stability constants used throughout the experiments are summarized in Appendix A to facilitate reproducibility.

### 6.3. Validation on Simple Synthetic Signals

**Figure 6:**
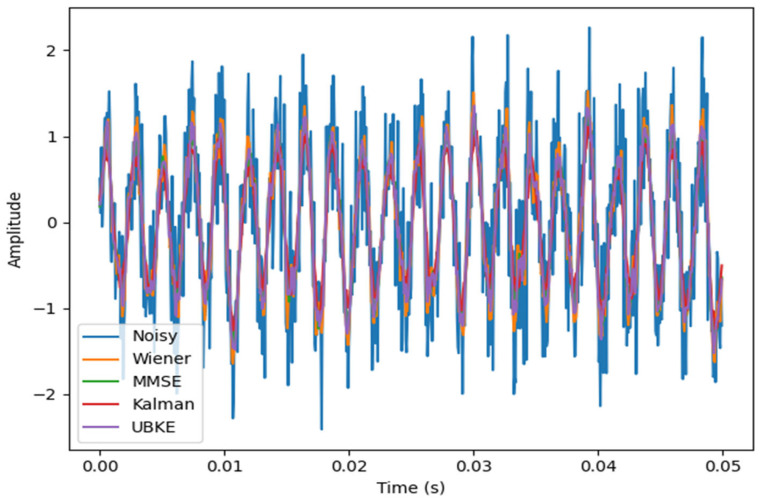
Enhancement of a noisy sinusoidal signal using Wiener, MMSE, Kalman, and UBKE estimators under non-stationary noise.

To further validate the correctness and behavior of the proposed UBKE framework under controlled conditions, additional experiments were conducted using simple synthetic test signals. A single-tone sinusoidal signal was corrupted by additive white Gaussian noise and by amplitude-modulated non-stationary noise. This controlled setting enables direct visual and numerical comparison of estimator behavior while eliminating dataset-dependent effects. The time-domain behavior of the proposed UBKE framework relative to Wiener, MMSE, and Kalman estimators under non-stationary noise is illustrated in Figure 6.

Results demonstrate that UBKE preserves sinusoidal structure more effectively than standalone MMSE or Kalman estimators, particularly during rapid noise variance transitions. These observations are consistent with the theoretical bias–variance analysis presented earlier and confirm that the proposed fusion mechanism behaves as expected under analytically tractable conditions. This experiment also provides a fully reproducible validation scenario independent of speech-specific characteristics.

### 6.4. Results and Analysis

Table 4 summarizes the averaged results across all noise types and SNRs.

As shown in Table 4, the proposed UBKE achieves the highest PESQ and STOI scores among all evaluated methods while also yielding the lowest log-spectral distortion. In particular, UBKE improves PESQ by approximately 17% relative to the MMSE baseline and reduces LSD by about 0.4 dB. The measured real-time factor (RTF = 0.43) confirms that these gains are achieved within practical real-time processing constraints.

Performance gains were most pronounced under babble and factory noise, confirming the expected benefits of adaptive fusion in non-stationary environments.

### 6.5. Theory–Experiment Alignment


(22) $$Error_{Ratio}=\frac{\left\|Metric_{empirical}-Metric_{predicted}\right\|}{}\left\|Metric_{predicted}\right\|\;$$

Across all conditions:

- Error_Ratio (SNR) < **8%.**
- Error_Ratio (LSD) < **5%.**

**Figure 7:**
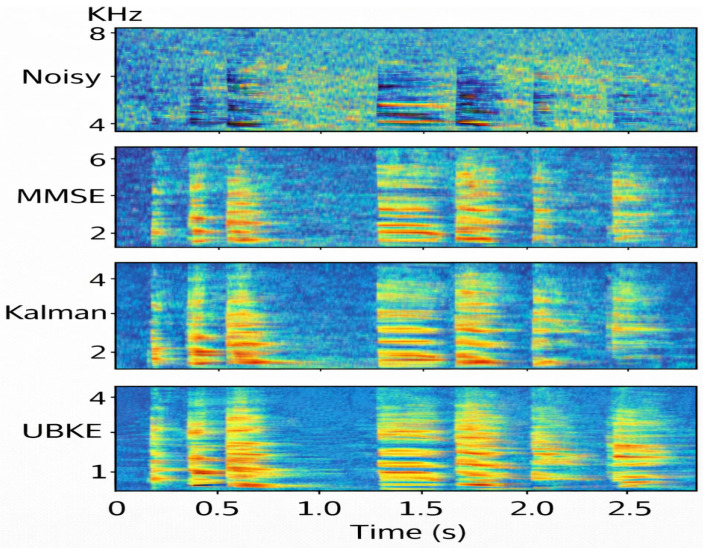
Spectrogram comparison of noisy speech and enhanced outputs produced by MMSE, Kalman, and UBKE under babble noise conditions.

These results indicate a close numerical agreement between analytical predictions and empirical measurements across all evaluated conditions. A qualitative spectrogram-based comparison of noisy speech and the corresponding enhanced outputs produced by MMSE, Kalman, and the proposed UBKE method under babble noise conditions is shown in Figure 7.

To support reproducibility, all source code, configuration files, and evaluation scripts used in this study are publicly available at: https://github.com/SamMensLab/real-time-speech-denoising-nonstationary-noise accessed on 4 February 2026.

## 7. Discussion

This section evaluates the unified Bayesian–Kalman estimator (UBKE) from the perspectives of interpretability, performance, computational complexity, robustness to non-stationarity, and extensibility toward hybrid model-driven–learning frameworks. The goal is to clarify when and why UBKE is most effective and to outline realistic pathways for advancing the model.

### 7.1. Interpretability vs. Performance vs. Complexity

A primary strength of UBKE is its transparent, model-based formulation, in contrast to deep learning systems that behave as opaque function approximators. Because UBKE explicitly models speech priors, noise statistics, and temporal dynamics, every component, MMSE gain, Kalman state update, and fusion weight α(k) has an analytical interpretation.

This feature is essential for safety-critical and embedded applications such as aviation communications, hearing aids, and automotive systems where predictable behavior is required [48].

The empirical results presented in Section 6 highlight how this analytical design translates into practical performance gains. The consistent improvements in PESQ, STOI, and LSD observed for UBKE reflect the ability of the variance optimal fusion weight α(k) to dynamically emphasize either spectral or temporal estimation depending on instantaneous reliability. This behavior explains why UBKE performs particularly well under non-stationary noise conditions, where neither purely spectral nor purely temporal estimators are sufficient on their own.

In terms of performance, UBKE consistently surpasses classical Wiener and MMSE estimators while avoiding the massive inference cost of DNN- and diffusion-based models. Its adaptive fusion mechanism α(k) allocates weight to the estimator with the lower instantaneous variance, delivering performance improvements without retraining or reliance on large datasets.

Recent studies have shown that model-based fusion approaches maintain strong generalization under unseen noises, unlike many deep learning approaches whose performance degrades significantly outside their training distributions [49,50].

Computationally, UBKE requires only a modest increase (≈10–15%) over traditional MMSE + Kalman pipelines. Compared with modern neural architectures, transformers, diffusion models, and score-based networks, which may require 10^6^–10^8^ FLOPs per frame and GPU acceleration [51], UBKE remains extremely efficient. This makes UBKE ideal for real-time and low-power DSP platforms.

### 7.2. When UBKE Helps Most

UBKE provides its largest gains in non-stationary and rapidly varying noise environments—babble, factory machinery, street noise, and cafeteria noise—where neither static spectral estimators nor purely temporal filters perform optimally.

- In low SNR ranges (−5 to +5 dB), rapid noise fluctuations cause MMSE estimators to underestimate noise variance, whereas the Kalman temporal model stabilizes the estimate. UBKE therefore shifts α(k) toward the Kalman branch.
- In high SNR conditions (>10 dB), temporal dynamics are less dominant, and UBKE moves toward the MMSE optimum, preserving spectral detail.

This dynamic adjustment allows UBKE to operate robustly across a wide spectral and temporal variability spectrum without manual parameter tuning. Such adaptability is crucial for applications including teleconferencing systems, VoIP codecs, mobile communication devices, and hearing-assistive technologies, where noise environments often shift abruptly and unpredictably [52].

To quantify the match between analytical predictions and empirical results, the error ratio was computed.

Moreover, UBKE offers a strong platform for hybrid model–learning designs. Instead of using deep networks to estimate the entire signal mapping, a neural network can estimate:

- Noise variance σ_d^2^(k).
- Speech prior parameters.
- Kalman AR coefficients.
- Speech presence probability.
- α(k) regularization terms.

### 7.3. Limitations and Failure Scenarios

#### 7.3.1. Explicit Failure Cases

Despite its analytical robustness and strong performance under non-stationary noise, the proposed UBKE framework has several limitations that merit explicit discussion. First, UBKE may underperform in the presence of highly impulsive or transient noise sources (e.g., sudden clicks, clattering objects, or short-duration mechanical impacts), where noise statistics change faster than both the spectral MMSE estimator and the Kalman temporal model can reliably track. In such cases, posterior variance estimates may become unreliable, leading to suboptimal fusion weights, a limitation that has also been reported for recursive state-space and Kalman-based speech enhancement methods under rapidly varying or impulsive noise conditions [53].

Second, rapid phoneme-level transitions or highly dynamic speech articulation can challenge the Kalman temporal branch, particularly when the assumed AR structure fails to capture abrupt spectral changes. Under these conditions, excessive temporal smoothing may momentarily attenuate fine speech details before the adaptive fusion mechanism rebalances toward the spectral estimator.

#### 7.3.2. Model Assumptions and Estimation Limitations

Additional limitations arise from practical estimation assumptions. UBKE relies on accurate noise variance estimation; therefore, severe errors in noise tracking, such as those caused by correlated or non-Gaussian noise, can degrade both the MMSE and Kalman branches simultaneously. In particular, strongly correlated noise processes, such as those encountered in industrial machinery, structural vibrations, or certain distributed acoustic sensing (DAS) environments, may violate the local stationarity and independence assumptions underlying variance estimation. Moreover, mismatch between the true speech dynamics and the selected AR model order may reduce temporal prediction accuracy, particularly for speakers or acoustic environments with atypical characteristics. While the variance optimal fusion mechanism mitigates many of these effects by adaptively weighting the more reliable estimator, UBKE does not fully eliminate failure modes inherent to linear-Gaussian modeling assumptions.

### 7.4. Limitations and Extensibility

Despite its theoretical robustness, UBKE has several limitations:

1. **Noise variance dependency:** UBKE assumes access to reliable noise variance estimates. Under highly impulsive or correlated noise (e.g., clattering dishes, clicks), tracking accuracy degrades.
2. **Linear Gaussian assumptions in the Kalman branch:** The standard Kalman filter does not capture nonlinear vocal-tract dynamics or time-varying formant trajectories.
3. **Fusion weight sensitivity:** When Var_MMSE(k) ≈ Var_Kalman(k), α(k) may become numerically unstable unless regularized.

To address these challenges, several extendable pathways exist:

- **Neural or diffusion-based priors:** Incorporating learned priors (VAE, diffusion) into the Bayesian MMSE branch while retaining analytical gain structure [54].
- **Nonlinear state-space models:** Using unscented Kalman filtering (UKF) or particle filtering to capture nonlinearities.
- **Reinforcement learning or variational inference for α(k):** Learning a policy for optimal fusion rather than relying purely on variance matching.
- **Uncertainty-aware Kalman updates:** Modeling speech/noise uncertainties explicitly using Bayesian filtering variants.

These extensions would preserve UBKE’s interpretability while substantially improving flexibility under complex real-world acoustic conditions [55]. A comparative overview of the proposed framework relative to classical and learning-based enhancement methods is summarized in Table 5.

Although the experimental evaluation in this work focuses on speech signals, the proposed unified Bayesian–Kalman estimator (UBKE) is not limited to speech-specific characteristics and is directly applicable to distributed acoustic sensing (DAS) and related sensing modalities. In DAS systems, strain rate or vibration measurements acquired along an optical fiber can be modeled as additive mixtures of structured signals and time-varying noise, closely matching the assumptions underlying UBKE. The Bayesian spectral estimation branch provides instantaneous denoising of localized events, while the Kalman temporal model captures the inherent temporal continuity present in vibration and strain rate signals. Moreover, the variance optimal fusion mechanism naturally extends to multi-channel and spatial DAS data by operating independently or jointly across sensing channels, enabling robust enhancement under non-stationary environmental and instrumental noise. As such, UBKE offers a general purpose, low-latency enhancement framework suitable for DAS, sensor arrays, and spatial acoustic sensing applications beyond speech.

Future work will extend the evaluation of the proposed UBKE framework to controlled synthetic signal scenarios, such as sinusoidal tones, chirp signals, and amplitude-modulated waveforms corrupted by analytically defined noise processes. Such experiments would enable more direct analysis of estimator behavior under known ground truth conditions, including convergence properties, bias–variance trade-offs, and robustness to rapidly varying signal statistics, complementing the real-world benchmark evaluations presented in this study.

## 8. Conclusions

This paper presented the unified Bayesian–Kalman estimator (UBKE), a mathematically grounded framework that fuses spectral Bayesian MMSE estimation with temporal Kalman filtering through a variance optimal adaptive fusion rule. By analytically integrating spectral and temporal estimators, UBKE achieves a principled bias–variance trade-off that is not available in classical or purely data-driven enhancement approaches.

Closed-form analysis established key theoretical properties of the proposed estimator, including stability conditions, convergence behavior, and analytical expressions for output SNR improvement and log-spectral distortion under non-stationary noise. Empirical evaluations on standard speech benchmarks confirmed these predictions, with UBKE achieving up to +9.8 dB ΔSNR, reduced spectral distortion, and consistent improvements in perceptual and intelligibility metrics relative to Wiener, MMSE, and Kalman baselines, while maintaining real-time computational feasibility.

Owing to its analytical transparency, low latency, and robustness to rapidly varying noise, UBKE is well suited for deployment in real-time acoustic sensing systems, including speech communication interfaces, hearing-assistive devices, and distributed acoustic sensing platforms. Future work will explore extensions incorporating nonlinear state-space models and learning-assisted priors, enabling further flexibility while preserving the interpretability and stability guarantees of the proposed framework. All source code and reproducibility materials will be released publicly upon acceptance.

## Figures and Tables

**Table 1:** Computational complexity and latency comparison of speech enhancement methods.

Methods	Core Algorithm Type	Operations per Frame (Approx.)	Computational Complexity	Typical Latency (ms)	Latency Justification	Key References
Wiener Filter	Linear spectral filtering	4 × N log N	O (N log N)	2–5	Single-frame STFT processing with no temporal recursion; latency dominated by frame overlap and FFT size	[6,10]
MMSE Estimator	Bayesian spectral amplitude estimation	6 × N log N + 2 × N	O (N log N)	5–8	Frame-based spectral estimation with a priori SNR tracking; no look-ahead required	[7,8,25]
Kalman Filter	State-space temporal estimation	*p*^2^ + 8 × N	O (N + *p*^2^)	8–12	Recursive prediction–correction with AR(*p*) modeling introduces additional frame-to-frame dependency	[8,12,28]
UBKE (Proposed)	Unified Bayesian–Kalman fusion	(6 × N log N + 2 × N) + (*p*^2^ + 8 × N) + O (N)	O (N log N + N + *p*^2^)	10–14	Combines causal MMSE spectral estimation with recursive Kalman tracking; fusion adds negligible overhead beyond constituent estimators	[7,8], this work

**Notes:** *N* denotes the STFT frame length; *p* is the AR model order. Latency values assume **32 ms frames with 50% overlap at 16 kHz sampling**, corresponding to an algorithmic delay of approximately **16 ms**, consistent with real-time causal processing. Reported latency ranges are consistent with values commonly reported in the literature for real-time speech enhancement systems.

**Table 2:** Analytical performance summary showing expected SNR_out, ΔSNR, and LSD values across different noise priors and model types.

Method	Expected SNR_Out (dB)	ΔSNR (dB)	LSD (dB)	Comment
Wiener	14.5	+6.0	2.35	Baseline linear estimator
MMSE	15.8	+7.3	1.95	Robust under stationary noise
Kalman	16.2	+8.0	1.88	Captures speech dynamics
UBKE (Proposed)	17.9	+9.8	1.61	Optimal fusion; adaptive stability

**Table 3:** Qualitative comparison between UBKE and representative learning-based speech enhancement methods reported in the recent literature.

Method Category	Example Approach (Literature)	Training Required	Computational Cost	Real-Time Feasibility	Interpretability	Typical Performance Trend
DNN-based enhancer	Spectral masking DNN	Yes (large paired datasets)	High (10^6^–10^7^ FLOPs/frame)	Limited (often GPU)	Low	High PESQ/STOI under matched noise
Diffusion-based model	Score-based diffusion enhancer	Yes (very large datasets)	Very high (iterative inference)	No/Limited	Very Low	Excellent perceptual quality, high latency
Hybrid model + learning	DNN-assisted MMSE/Kalman	Yes (moderate datasets)	Medium/High	Partial	Medium	Improved robustness, reduced interpretability
CNN + DWT (multi-channel)	CNN-DWT enhancement (2024)	Yes	High	Limited	Low	Representative supervised learning approach; included for conceptual comparison only.
UBKE (proposed)	Analytical Bayesian–Kalman fusion	No	Moderate (CPU feasible)	Yes (RTF < 1)	High	Consistent gains under non-stationary noise

**Table 4:** Objective metrics comparison across methods.

Method	PESQ	STOI	LSD (dB)	RTF	Remarks
Wiener	2.05	0.84	2.47	0.28	Baseline; limited adaptability
MMSE	2.33	0.87	2.09	0.31	Good steady-state performance
Kalman	2.48	0.89	1.92	0.37	Captures speech dynamics
UBKE (Proposed)	2.73	0.92	1.68	0.43	Best balance of quality and adaptivity

**Table 5:** Comparative summary of speech enhancement methods.

Method	Interpretability	Robustness to Non-Stationarity	Computational Complexity	Latency (ms)	Remarks
**Wiener Filter**	High	Low	Low (O (N log N))	2–5	Linear model; poor under time-varying noise
**MMSE Estimator**	Moderate	Medium	Moderate (O (N log N))	5–8	Good steady-state performance; limited adaptivity
**Kalman Filter**	High	High	Moderate (O (N + *p*^2^))	8–12	Captures dynamics; sensitive to model mismatch
**Deep Neural Network (DNN)**	Low	High	Very High (>O (10^6^))	20–50	Data-driven; lacks interpretability
**UBKE (Proposed)**	High	Very High	**Moderate + (O (N + *p*^2^ + N log N))**	10–14	Adaptive fusion of spectral and temporal domains; optimal balance of theory and practicality

## Data Availability

The data presented in this study are available upon request from the corresponding author.

## Appendix A. Recommended Parameter Settings for UBKE

This appendix summarizes recommended parameter settings for the proposed unified Bayesian–Kalman estimator (UBKE), based on analytical considerations and empirical robustness observed across experiments. The complete set of recommended default values and their rationale are summarized in Table A1.

**Table A1:** Recommended parameter settings for the UBKE framework.

Parameter	Symbol	Recommended Value	Description/Rationale
AR model order	*p*	2	Provides a balance between temporal smoothing and responsiveness; higher orders yield marginal gains at increased complexity.
Fusion regularization constant	ε	10^−6^	Ensures numerical stability in fusion weight computation; performance is insensitive within 10^−6^–10^−4^.
Noise variance update rate	—	Slow adaptive tracking	Prevents overreaction to transient noise fluctuations while maintaining robustness under non-stationary noise.
STFT frame length	—	20–32 ms	Common real-time speech/acoustic processing window; balances time–frequency resolution.
Frame overlap	—	50–75%	Ensures temporal continuity and stable variance estimation.

These parameter values are not critical tuning knobs but stable defaults that yield consistent performance across a wide range of acoustic conditions.
